# Effects of Lateral Positions in Second‐Stage Labour on Maternal and Neonatal Outcomes: An Observational Study Using Propensity Score Matching Analysis

**DOI:** 10.1111/ijn.70114

**Published:** 2026-02-04

**Authors:** Jing Huang, Hong Lu, Jianying Wang, Minghui Yang, Yinchu Hu, Xue Feng, Lihua Ren, Yu Zang, Hou Rui

**Affiliations:** ^1^ Florence Nightingale Faculty of Nursing, Midwifery and Palliative Care King''s College London London UK; ^2^ School of Nursing Peking University Beijing China; ^3^ Labour Room, Northwest Women's and Children's Hospital Xian China; ^4^ First Affiliated Hospital of Kunming Medical University Kunming China; ^5^ School of Nursing Hebei Medical University Shijiazhuang China

**Keywords:** childbirth, labour, lateral position, midwifery, nursing

## Abstract

**Aim:**

This study aims to examine the effects of lateral positions during the second stage of labour on maternal and neonatal outcomes.

**Design:**

A prospective observational study was conducted in three Chinese hospitals from June to November 2020.

**Methods:**

Eligible women were divided into groups based on their use of lithotomy or lateral positions during the second stage of labour. Propensity score matching (PSM) was employed to assess the effects of lateral positions on maternal outcomes (perineal tears, episiotomy use, postpartum blood loss, duration of the second stage of labour, and mode of birth) and neonatal outcomes (Apgar scores at one and 5 min postpartum).

**Results:**

After PSM matching, lateral positions were associated with a 21% reduced risk of episiotomy use among primiparous women. Primiparous women adopting lateral positions also had significantly reduced risks of moderate perineal tears. There were no significant differences in the duration of the second stage of labour, mode of birth or rates of blood loss over 500 mL at 2 h postpartum. However, lateral positions increased the duration of the second stage of labour by 10 min among primiparous women who adopted lateral positions in this study, and both primiparous and parous women using lateral positions had greater postpartum blood loss. Cases of Apgar scores less than seven and severe perineal tears were rare.

**Conclusion:**

This prospective observational study indicates that lateral positions during the second stage of labour may offer protective benefits for the perineum among primiparous women. However, their use requires careful clinical judgement, as they may not be suitable for primiparous women experiencing slower labour progress, and additional attention should be given for monitoring postpartum blood loss, regardless of parity.

## Summary

What is already known?

- Maternal positions during the second stage of labour are closely associated with maternal and neonatal outcomes.
- In recent years, the adoption of lateral positions during this stage has gained popularity in China and could play a crucial role in promoting safe vaginal births. Nevertheless, the effects of these positions on maternal and neonatal outcomes remain uncertain.

What this paper adds?

- Lateral positions during the second stage of labour may prevent perineal tears, with greater protective impacts observed among primiparous women who actually adopted lateral positions in clinical practice.
- Lateral positions may increase the amount of postpartum blood loss, regardless of parity.
- Lateral positions may prolong the second stage of labour among primiparous women.

What are the implications of this study?

- Findings from this study suggest that while lateral positions during the second stage of labor may offer benefits, such as reducing perineal tears, they also carry potential risks, including increased postpartum blood loss and a longer second stage of labour for primiparous women.
- Providing individualized care that considers a woman's parity and preferences is essential for optimizing maternal and neonatal outcomes.

## 1 Introduction

Maternal positions during the second stage of labour are closely associated with maternal and neonatal outcomes (Zang, Lu, Zhao, et al. 2020). In China, the lithotomy position is commonly used, despite substantial evidence indicating potential negative impacts on these outcomes (Huang et al. 2019). Recently, there has been a growing trend in China towards the use of lateral positions during the second stage of labour. However, the effects of lateral positions on maternal and neonatal outcomes remain unclear due to a lack of high‐quality research focusing on this aspect. Moreover, given the increasing adoption of lateral positions in clinical practice, there is a need for more detailed guidance on their appropriate use in different populations. Therefore, further research is needed.

### 1.1 Background

Childbirth is a major life event for women and their families (Kopas 2014). The second stage of labour, defined as the time from full cervical dilation to the birth of the foetus (Cunningham et al. 2018), is the most critical and stressful phase of childbirth. Maternal positions during this phase constitute an important part of childbirth management (Huang et al. 2019). They play a pivotal role in safe childbirth, as they may influence maternal and neonatal outcomes by affecting aortocaval compression, uterine contractions, pelvic dimensions, foetal head moulding and the progression of foetal head angle through the birth canal (Gupta et al. 2017; Kibuka et al. 2021). However, there is currently no consensus on the most appropriate maternal position during this stage of labour.

Two main classifications are used to categorize maternal positions. Atwood's classification identifies two groups: upright positions and horizontal positions, based on the angle formed by the line connecting the centres of a woman's third and fifth lumbar vertebrae and the horizontal plane (Atwood 1976). Upright positions include squatting, standing, sitting and kneeling positions, whereas horizontal positions typically refer to lithotomy positions, supine and lateral positions.

To date, most studies have compared the effects of upright positions and lithotomy positions on maternal and neonatal outcomes. Upright positions had been recommended by several guidelines due to their potential benefits in shortening the second stage of labour, reducing abnormal foetal heart rate patterns and promoting spontaneous vaginal births (Huang et al. 2019; Zang, Lu, Zhang, et al. 2020). However, some upright positions also carry potential risks, including perineal trauma and a greater risk of postpartum blood loss greater than 500 mL (Haslinger et al. 2015; Thies‐Lagergren et al. 2011). Additionally, limited midwifery staffing and safety concerns may hinder the use of upright positions in clinical practice (Zang et al. 2021).

Another system classifies maternal positions into flexible sacrum positions and non‐flexible sacrum positions (Kemp et al. 2013). Sacrum positions remove pressure from the sacrum. Under this system, lateral positions, along with other common upright positions, fall into the flexible sacrum category. A systematic review found that flexible sacrum positions are associated with a higher rate of spontaneous vaginal childbirth, lower rates of episiotomy use and severe perineal trauma and a shorter duration of the active pushing phase (Zang, Lu, Zhao, et al. 2020). However, this review also noted that women adopting flexible sacrum positions may have a higher risk of mild perineal trauma.

In China, the lithotomy positions are frequently used during the second stage of labour, despite evidence showing their negative impacts on both the maternal and neonatal outcomes (Huang et al. 2019). A national survey found that nearly half of the hospitals in China do not support a free choice of other maternal positions during the second stage of labour (Lu et al. 2020). Other studies have reported similar findings (Wu et al. 2021; Yang et al. 2020). In recent years, however, lateral positions have become more commonly adopted. A provincial survey reported that 30% of hospitals had begun supporting lateral birth positions (Dou et al. 2018). Nevertheless, the effects of lateral positions on maternal and neonatal outcomes among Chinese women remain unclear.

Lateral positions are a type of horizontal position and also belong to the flexible sacrum position category. Biomechanically, they may improve maternal and neonatal outcomes compared with the lithotomy positions. For example, lateral positions may facilitate pelvic outlet expansion, potentially helping the foetal head to pass more easily through the birth canal, thereby shortening the second stage of labour. Intrauterine pressure might be reduced in lateral positions (Simkin et al. 2017), helping to maintain adequate foetal blood supply and potentially reducing the risk of neonatal asphyxia. Additionally, lateral positions can provide midwives with easier access to monitor labour progress and intervene when necessary.

To date, high‐quality evidence on the effects of lateral positions among Chinese women is limited due to methodological issues, such as failure to control for confounding effects and unclear outcome definitions. Data on the use of lateral positions among parous women are also limited, as most studies have not differentiated between primiparous and parous women. Existing studies have reported mixed results. The safety and feasibility of lateral positions in clinical practice require further investigation.

### 1.2 Objectives

This study aimed to analyse the effects of lateral positions during the second stage of labour on the rates of perineal tears, episiotomy use, duration of the second stage of labour, blood loss at 2 h postpartum, mode of birth and Apgar scores at 1 min and 5 min among the Chinese population.

## 2 Method

### 2.1 Study Design

This was a prospective observational study involving both primiparous and parous Chinese women giving birth in three tertiary hospitals. These hospitals had recently begun to support women adopting lateral positions during the second stage of labour. The reporting of this study followed Strengthening the Reporting of Observational Studies in Epidemiology (STROBE) guidelines (von Elm et al. 2014).

### 2.2 Setting

Three tertiary hospitals were conveniently selected due to the feasibility of conducting the current study. Hospital A and Hospital B are both located in Kunming, Yunnan province. Specifically, Hospital A is a general hospital handling different kinds of diseases and injuries, with approximately 6700 births annually. Hospital B is a specialized tertiary hospital providing maternal and children's care in Yunnan province, with an estimated 5800 births per year. Hospital C, the largest specialized maternal and child care hospital in Xi'an, Shaanxi province, has approximately 7000 births yearly.

The three hospitals have begun to support women giving birth in lateral positions during the second stage of labour recently. Midwives working in these hospitals received relevant training on the use of lateral positions during both the passive and active phases of the second stage of labour. Decisions on maternal positions were made by midwives and women together. The commonly adopted maternal position in these hospitals was the lithotomy position.

### 2.3 Participants

The eligibility criteria of participants in this study were (1) singleton pregnancy; (2) a gestational age from 37^+0^ to 42^+0^ weeks; (3) cephalic presentation; (4) age over 18 years; (5) adoption of lateral or lithotomy positions during both the passive and active phases of the second stage of labour. Women were excluded from this study if they had (1) serious pregnancy complications, including gestational diabetes and pregnancy‐induced hypertension; (2) serious childbirth complications, such as shoulder dystocia, uterine rupture or amniotic fluid embolism; and (3) a planned Caesarean section.

### 2.4 Outcomes and Measurements

The primary outcomes of this study were the rates of perineal tears and episiotomy.

Perineal tears were categorized as intact perineum, first‐degree perineal tears, second‐degree perineal tears and third‐/fourth‐degree perineal tears. As first‐degree perineal tears usually do not require suturing and are associated with fewer negative outcomes, they can be regarded as one of the ideal outcomes for women. Thus, rates of first‐degree perineal tears and intact perineum were grouped and referred to as mild perineal tears in this study. Because second‐degree perineal tears involve injuries similar to those caused by episiotomy, and both require suturing in clinical practice, the rates of episiotomy and second‐degree perineal tears were combined and referred to as moderate perineal tears.

Two midwives assessed the degree of perineal tears according to the classification of the Royal College of Obstetricians and Gynaecologists (RCOG) (Royal College of Obstetricians and Gynaecologists 2015). Given that third‐ and fourth‐degree perineal tears are generally considered serious adverse events, they were assessed jointly by obstetricians and midwives. The rates of third‐ and fourth‐degree perineal tears were combined and referred to as severe perineal tears in this study.

The indications for episiotomy use were in accordance with the midwifery textbook used in China (Yu and Chen 2017). Two midwives made decisions regarding episiotomy use jointly.

The secondary outcomes of this study included blood loss at 2 h postpartum, duration of the second stage of labour, mode of birth, and neonatal Apgar scores at one and 5 min postpartum. Blood loss at 2 h postpartum was selected because it is standard practice to record this in the participating hospitals. Midwives measured the amount of blood loss by weighing pads, sheets and other textiles and by measuring the volume of blood collected in a pan. The proportion of women with blood loss exceeding 500 mL at 2 h postpartum was calculated as an outcome of interest to better assess the safety of lateral positions.

Similarly, midwives recorded the duration of the second stage of labour, defined as the time from full cervical dilation to the birth of the baby. The rates of prolonged second stage of labour among women adopting lateral and lithotomy positions were presented in this study. A prolonged second stage of labour was defined as no progress in descent or rotation for more than 4 h in primiparous women with epidural analgesia, more than 3 h in primiparous women without epidural analgesia, more than 3 h in parous women with epidural analgesia or more than 2 h in parous women without epidural analgesia (Yu and Chen 2017).

Modes of birth included spontaneous vaginal birth and assisted vaginal birth (vacuum to forceps births). Neonatal Apgar scores at 1 and 5 min after birth were assessed by attending midwives. As an Apgar score below seven may indicate a higher risk of complications (Watterberg et al. 2015), it was considered an outcome of interest to evaluate newborn status.

### 2.5 Sample Size Calculation

The rate of moderate perineal tears was chosen as the indicator for sample size calculation due to its clinical significance. G*Power was used to determine the sample size (Faul et al. 2009). A priori power analysis was performed based on the difference in rates of moderate perineal tears between primiparous women adopting lateral positions and those in lithotomy positions during the second stage of labour. The rates of moderate perineal tears were obtained from a previous study (Wei et al. 2017). The required sample size for primiparous women was 86, given a p_1_ of 17.9%, p_2_ of 54.0%, statistical power of 0.95, and a Type I error of 0.05.

Because data on the rates of moderate perineal tears among parous women adopting lateral positions and lithotomy positions are scarce, and because nulliparity is a known risk factor for perineal tears (Hu et al. 2022), we assumed that the minimal sample size for parous women should also be 86. Considering a possible 10% of missing data, the total sample size was increased to 190.

### 2.6 Data Collection

A pre‐designed data collection form was developed by the research team to collect the following information:

1. General information: Maternal age, date of birth, parity, height, pre‐pregnancy weight, weight at childbirth and Caesarean history.
2. Maternal positions: Lithotomy positions or lateral positions during both the passive and active phases of the second stage of labour.
3. Obstetric information: Mode of birth, labour induction, foetal position, epidural analgesia use, labour augmentation (oxytocin use), duration of the second stage of labour, perineal tears, episiotomy use and amount of blood loss at 2 h postpartum.
4. Newborn information: Birth weight and Apgar scores at 1 and 5 min after childbirth.

Additionally, midwives received a training programme and detailed instructions on data collection procedures to ensure data quality.

Women giving birth in three selected hospitals between July and November 2020 were approached and assessed for eligibility by attending midwives on a daily basis. Midwives completed the data collection form after each birth. All the data collection forms were mailed to the researcher (J.H.).

### 2.7 Ethical Considerations

The present study received approval from the Institutional Review Board of Peking University (IRB approval Number: IRB00001052‐2004). Institutional permits were also obtained from the three hospitals involved. Midwives explained the study to eligible women prior to enrolment. Written informed consent was obtained. Participation in this study was voluntary, and participants were free to withdraw at any time. The confidentiality of all data was strictly maintained.

### 2.8 Statistical Methods

In this study, categorical variables were presented as counts and frequencies, whereas continuous variables were expressed as either means (standard deviations) or medians (with inter‐quartile ranges), depending on the normality of data distribution.

To reduce the influence of observed confounding variables on group allocation and outcomes, propensity score matching (PSM) was applied to obtain matched groups. The propensity score in this study represented women's probability of adopting lateral positions during the second stage of labour. In the participating hospitals, the choice of maternal position was generally based on women's preferences and the attending midwives' judgement. Factors influencing midwives' decisions included maternal age (years), vaginal birth after Caesarean section (VBAC) (yes/no), epidural analgesia use (yes/no), cephalic position (occiput anterior vs. other cephalic presentations), oxytocin augmentation (yes/no), pre‐pregnancy BMI (kg/m^2^), neonatal birthweight (g) and labour induction (yes/no).

PSM analysis was performed using a 1:1 matching algorithm with replacement, based on the above observable covariates. Matching with replacement was chosen to ensure well‐matched groups despite a relatively small sample size. The calliper width was set at 0.20 of the standard deviation of the logit of the propensity score (Austin 2014).

Post‐matching balance was assessed by examining mean differences between the treatment and the control groups using percentage of bias, variance ratios and *p*‐values from the *t*‐test of covariates (Zhang et al. 2019). Balanced was considered acceptable if the percentage of bias was below 10%, variance ratios approached 1 or below 2, and *t*‐tests were non‐significant (Zhang et al. 2019).

After matching, average treatment effects (ATE) and average treatment effects on the treated (ATT) were estimated to assess the effects of lateral positions. ATE refers to the average difference between observed and potential outcomes for each individual, regardless of the treatment (lateral positions). ATT refers specifically to the average treatment effect among those who actually adopted lateral positions. It is calculated as the difference between:

- the observed maternal and neonatal outcomes of women who adopted lateral positions; and
- their potential outcomes had they adopted the lithotomy positions (Greifer and Stuart 2021).

Two key assumptions underpin PSM analysis: unconfoundedness and overlap. Unconfoundedness implies that, after controlling for observable covariates, potential outcomes are independent of treatment assignment (maternal positions). The overlap assumption indicates that, for every set of covariates X, individuals have a positive and non‐zero probability of being assigned to either the treatment or control group (lateral or lithotomy positions) (Li 2013). These two assumptions must be satisfied to ensure strong ignorability (Rosenbaum and Rubin 1983).

All statistical analyses were performed using STATA 17.0. A two‐sided *p*‐value of less than 0.05 was considered statistically significant.

## 3 Results

### 3.1 Characteristics of Participants

A total of 848 women participated in this study, of whom 52.5% (*n* = 445) were primiparous, and 47.5% were parous (*n* = 403). Overall, 25.4% of participants adopted lateral positions during both the passive and active phases of the second stage of labour, whereas 74.6% remained in the lithotomy positions.

Table 1 presents the baseline and pregnancy characteristics of women who adopted lateral versus lithotomy positions during the second stage of labour. Compared with those in the lithotomy group, women who gave birth in lateral positions were less likely to receive oxytocin augmentation and more likely to receive epidural analgesia, regardless of parity. Additionally, parous women who assumed lateral positions tended to have a higher pre‐pregnancy BMI compared to those who gave birth in lithotomy positions.

**TABLE 1:** Baseline and pregnancy characteristics of women adopting lateral and lithotomy positions during the second stage of labour (*n* = 848).

	Primiparous women (*n* = 445)			Parous women (*n* = 403)			All (*n* = 848)		
	Lateral positions (*n* = 118)	Lithotomy positions (*n* = 327)	P	Lateral positions (*n* = 98)	Lithotomy positions (*n* = 305)	P	Lateral positions (*n* = 216)	Lithotomy positions (*n* = 632)	*p*
Maternal age (years)									
< 25	21 (17.8%)	34 (10.4%)	0.08^a^	8 (8.2%)	16 (5.3%)	0.27^b^	29 (13.4%)	50 (7.9%)	0.04^b^ ^,^ *
25–34	92 (78.0%)	283 (86.6%)		80 (81.6%)	235 (77.1%)		172 (79.6%)	518 (82.0%)	
≥ 35	4 (3.4%)	8 (2.4%)		10 (10.2%)	47 (15.4%)		14 (6.5%)	55 (8.7%)	
Missing data	1 (0.8%)	2 (0.6%)		0 (0%)	7 (2.3%)		1 (0.5%)	9 (1.4%)	
Body mass index (kg/m^2^)									
< 18.5	16 (13.6%)	58 (17.7%)	0.11^b^	5 (5.1%)	49 (16.1%)	0.02^b^ ^,^ *	21 (9.7%)	107 (16.9%)	0.007^b^ ^,^ **
18.5–23.8	83 (70.3%)	225 (68.8%)		75 (76.5%)	203 (66.6%)		158 (73.2%)	428 (67.7%)	
≥ 23.9	19 (16.1%)	30 (9.2%)		17 (17.4%)	40 (13.1%)		36 (16.7%)	70 (11.1%)	
Missing data	0 (0%)	14 (4.3%)		1 (1.0%)	13 (4.3%)		1 (0.5%)	27 (4.3%)	
VABC									
Yes	—	—	—	93 (94.9%)	297 (97.4%)	0.15^a^	5 (2.3%)	6 (9.5%)	0.16^a^
No	—	—		5 (5.1%)	6 (2.0%)		211 (97.7%)	624 (98.7%)	
Missing				0 (0%)	2 (0.7%)		0 (0%)	2 (0.3%)	
Augmentation (oxytocin use)									
Yes	31 (26.3%)	126 (38.5%)	0.03*	27 (27.6%)	119 (39.0%)	0.05^b^ ^,^ *	58 (26.8%)	245 (38.8%)	0.003^b^ ^,^ **
No	79 (66.9%)	189 (57.8%)		65 (66.3%)	172 (56.4%)		144 (66.7%)	361 (57.1%)	
Missing	8 (6.8%)	12 (3.7%)		6 (6.1%)	14 (4.6%)		14 (6.5%)	26 (4.1%)	
Epidural analgesia									
Yes	108 (91.5%)	189 (57.8%)	< 0.001***	95 (96.9%)	238 (77.3%)	< 0.001^b^ ^,^ ***	11 (5.1%)	201 (31.8%)	< 0.001^b^ ^,^ ***
No	9 (7.6%)	136 (41.6%)		2 (2.0%)	65 (21.3%)		203 (94.0%)	425 (67.3%)	
Missing	1 (0.9%)	2 (0.6%)		1 (1.0%)	4 (1.3%)		2 (0.9%)	6 (0.9%)	
Cephalic presentation									
Occiput anterior	113 (95.8%)	319 (97.6%)	0.73^a^	96 (97.9%)	301 (98.7%)	0.57^a^	209 (96.8%)	620 (98.1%)	0.75^a^
Other cephalic presentations	3 (2.5%)	7 (2.1%)		1 (1.0%)	2 (0.7%)		4 (1.9%)	9 (1.4%)	
Missing data	2 (1.7%)	1 (0.3%)		1 (1.0%)	2 (0.7%)		3 (1.4%)	3 (0.5%)	
Birthweight (g)									
< 4000	114 (96.6%)	314 (96.0%)	0.68^a^	94 (95.9%)	285 (93.4%)	1.00^a^	208 (96.3%)	599 (94.8%)	0.43^a^
≥ 4000	1 (0.9%)	7 (2.1%)		2 (2.0%)	9 (2.9%)		3 (1.4%)	16 (2.5%)	
Missing data	3 (2.5%)	6 (1.8%)		2 (2.0%)	11 (3.6%)		5 (2.3%)	17 (2.7%)	
Induction of labour									
Yes	100 (84.7%)	266 (81.4%)	0.31^b^	89 (87.2%)	266 (87.2%)	0.32^b^	25 (11.6%)	97 (15.4%)	0.18^b^
No	17 (14.4%)	61 (18.7%)		8 (8.2%)	36 (11.8%)		189 (87.5%)	532 (84.2%)	
Missing	1 (0.9%)	0 (0%)		1 (1.0%)	3 (1.0%)		2 (0.9%)	3 (0.5%)	

Abbreviation: VABC, vaginal birth after Caesarean.

^a^ Fisher's exact test.

^b^ Chi‐square test.

*** *p* < 0.001.

** *p* < 0.01.

* *p* < 0.05.

### 3.2 Matching Results of PSM

A total of 98 primiparous and 84 parous women in the lateral position groups, and 287 primiparous and 257 parous women in the lithotomy position groups, provided the data for the counterfactual framework of causal inference analysis. The detailed matching assignment of observations of common support is shown in Table 2.

**TABLE 2:** Matching assignment of observations of common support.

Parity	Treatment assignment	Common support		Total
		Off support	On support	
Primiparous women	Lithotomy positions	48	239	287
	Lateral positions	0	98	98
Parous women	Lithotomy positions	19	238	257
	Lateral positions	0	84	84

As shown in Table 3, the percentage of bias was below 10% for all covariates in the matched groups. The *p*‐values of *t*‐tests were all non‐significant (*p* > 0.05), and the available variance ratios ranged from 0.87 to 1.48. These parameters indicate balanced covariate distribution between lateral and lithotomy position groups in this study.

**TABLE 3:** Baseline and pregnancy characteristics of women adopting lateral positions and lithotomy positions during the second stage of labour and risk of bias before and after propensity score matching.

Variables	Primiparous women (*n* = 445)						Parous women (*n* = 403)					
	Unmatched groups			Matched groups			Unmatched groups			Matched groups		
	Lateral positions (*n* = 118)	Lithotomy positions (*n* = 327)	Bias (%)	Bias (%)	*p*	Var(T)/Var(C)	Lateral positions (*n* = 98)	Lithotomy positions (*n* = 305)	Bias (%)	Bias (%)	*p*	Var(T)/Var(C)
Maternal age (years)												
< 25	21 (17.8%)	34 (10.4%)	−14.2%	2.6%	0.862	1.22	8 (8.2%)	16 (5.3%)	−20.6%	2.6%	0.870	0.95
25–34	92 (78.0%)	283 (86.6%)					80 (81.6%)	235 (77.1%)				
≥ 35	4 (3.4%)	8 (2.4%)					10 (10.2%)	47 (15.4%)				
Missing data	1 (0.8%)	2 (0.6%)					0 (0%)	7 (2.3%)				
Body mass index (kg/m^2^)												
< 18.5	16 (13.6%)	58 (17.7%)	29.7%	−0.0%	1.000	0.93	5 (5.1%)	49 (16.1%)	31.9%	−2.3%	0.872	0.87
18.5–23.8	83 (70.3%)	225 (68.8%)					75 (76.5%)	203 (66.6%)				
≥ 23.9	19 (16.1%)	30 (9.2%)					17 (17.4%)	40 (13.1%)				
Missing data	0 (0%)	14 (4.3%)					1 (1.0%)	13 (4.2%)				
VABC												
Yes	—	—	—	—	—	—	93 (94.9%)	297 (97.4%)	18.3%	6.8%	0.702	—
No	—	—					5 (5.1%)	6 (2.0%)				
Missing	—	—					1 (0.9%)	2 (0.7%)				
Augmentation (oxytocin use)												
Yes	31 (26.3%)	126 (38.5%)	24.3%	−6.5%	0.632	1.07	27 (27.6%)	119 (39.0%)	27.8%	−5.1%	0.728	1.06
No	79 (66.9%)	189 (57.8%)					65 (66.3%)	172 (56.4%)				
Missing	8 (6.8%)	12 (3.7%)					6 (6.1%)	14 (4.6%)				
Epidural analgesia												
Yes	108 (91.5%)	189 (57.8%)	−91.8%	−0.0%	1.000	—	95 (96.9%)	238 (77.3%)	−55.9%	−0.0%	1.000	—
No	9 (7.6%)	136 (41.6%)					2 (2.0%)	65 (21.3%)				
Missing	1 (0.9%)	2 (0.6%)					1 (1.0%)	4 (1.3%)				
Cephalic presentation												
Occiput anterior	113 (95.8%)	319 (97.6%)	6.1%	6.4%	0.653	1.48	96 (97.9%)	301 (98.7%)	9.0%	0.0%	1.000	1.00
Other cephalic presentations	3 (2.5%)	7 (2.1%)					1 (1.0%)	2 (0.7%)				
Missing data	2 (1.7%)	1 (0.3%)					1 (1.0%)	2 (0.7%)				
Birthweight (g)												
< 4000	114 (96.7%)	314 (96.0%)	−10.9%	0.0%	1.000	1.00	94 (95.9%)	285 (93.4%)	−15.3%	0.0%	1.000	1.00
≥ 4000	1 (0.9%)	7 (2.1%)					2 (2.0%)	9 (2.9%)				
Missing data	3 (2.5%)	6 (1.8%)					2 (2.0%)	11 (3.6%)				
Induction of labour												
Yes	100 (84.7%)	266 (81.4%)	−12.7%	0.0%	1.000	—	89 (87.2%)	266 (87.2%)	−11.4%	4.5%	0.734	—
No	17 (14.4%)	61 (18.7%)					8 (8.2%)	36 (11.8%)				
Missing	1 (0.9%)	0 (0%)					1 (1.0%)	3 (1.0%)				

Abbreviation: VABC, vaginal birth after Caesarean.

### 3.3 ATE and ATT of Lateral Positions on Primary Outcomes

Among primiparous women, if both groups had used lateral positions, the rate of episiotomy would have decreased by 21.49% (ATE: −21.49%, 95% CI: −35.84%, −7.15%; *p* = 0.003). Lateral positions also contributed to a non‐significant increase in mild perineal tears (ATE: 13.59%, 95% CI: −1.85%, 29.05%; *p* = 0.085) and a corresponding non‐significant reduction in moderate perineal tears (ATE: −13.59%, 95% CI: −29.05%, 1.85%; *p* = 0.085).

The protective effect of lateral positions on the perineum was more pronounced among primiparous women who actually adopted the lateral position, as demonstrated by a significantly reduced risk of moderate perineal tears (ATT: −14.51%, 95% CI: −26.89%, −2.13%; *p* = 0.022) and a notable increase in mild perineal tears (ATT: 14.52%, 95% CI: 2.13%, 26.89%; *p* = 0.022).

Lateral positions did not significantly affect rates of perineal tears or episiotomy use among the parous population (*p* > 0.05). No cases of severe perineal tears were observed among either primiparous or parous women in this study; therefore, relevant statistical analyses could not be conducted. Detailed results are presented in Tables 4 and 5.

**TABLE 4:** Average treatment effects (ATE) and average treatment effects on the treated (ATT) of lateral positions during the second stage of labour on maternal and neonatal outcomes in primiparous women using propensity score matching.

Outcomes	Maternal positions	Unmatched (%/mean ± SD)	Average treatment effects (ATE)			Average treatment effects on treated (ATT)		
			Coefficient (95% CI)	*z*	*p*	Coefficient (95% CI)	*z*	*p*
Mild perineal tears (%)	Lateral position	77.39%	13.59% (−1.85%, 29.05%)	1.72	0.085	14.52% (2.13%, 26.89%)	2.30	0.022*
	Lithotomy position	54.29%						
Moderate perineal tears (%)	Lateral position	22.61%	−13.59% (−29.05%, 1.85%)	−1.72	0.085	−14.51% (−26.89%, −2.13%)	−2.30	0.022*
	Lithotomy position	45.71%						
Severe perineal tears (%)	Lateral position	0%	—	—	—	—	—	—
	Lithotomy position	0%						
Episiotomy (%)	Lateral position	12.82%	−21.49% (−35.84%, −7.15%)	−2.94	0.003**	−23.49% (−34.69%, −12.29%)	−4.11	< 0.001***
	Lithotomy position	43.87%						
2‐h postpartum bleeding (mL)	Lateral position	298.64 ± 67.94	73.09 (44.84, 101.34)	5.07	< 0.001***	68.15 (48.40, 87.89)	6.76	< 0.001***
	Lithotomy position	228.57 ± 121.06						
2‐h postpartum bleeding > 500 mL (%)	Lateral position	1.82%	−0.40% (−3.34%, 2.55%)	−0.27	0.790	−1.00% (−3.82%, 1.81%)	−0.70	0.481
	Lithotomy position	2.48%						
Spontaneous vaginal birth (%)	Lateral position	99.14%	−1.68% (−7.36%, 4.00%)	−0.58	0.563	−0.10% (−2.74%, 2.94%)	0.07	0.944
	Lithotomy position	94.17%						
Duration of second stage of labour (min)	Lateral position	48.52 ± 31.01	1.65 (−10.05, 13.34)	0.28	0.783	10.39 (2.66, 18.12)	2.63	0.008**
	Lithotomy position	49.41 ± 35.31						
Prolonged second stage of labour (%)	Lateral position	2.56%	0.40% (−1.50%, 2.31%)	0.41	0.680	1.26% (−1.57%, 4.1%)	0.87	0.383
	Lithotomy position	0.60%						
Neonatal outcomes								
Apgar score < 7 (1 min)	Lateral position	0%	—	—	—			
	Lithotomy position	0%						
Apgar score < 7 (5 min)	Lateral position	0%	—	—	—			
	Lithotomy position	0%						

*** *p* < 0.001.

** *p* < 0.01.

* *p* < 0.05.

**TABLE 5:** Average treatment effects (ATE) and average treatment effects on the treated (ATT) of lateral positions during the second stage of labour on maternal and neonatal outcomes among parous women using propensity score matching.

Outcomes	Maternal positions	Unmatched (%/mean ± SD)	Average treatment effects (ATE)			Average treatment effects on treated (ATT)		
			Coefficient (95% CI)	*z*	*p*	Coefficient (95% CI)	*z*	*p*
Mild perineal tears (%)	Lateral position	88.66%	6.37% (−2.23, 14.97%)	1.45	0.146	6.01% (−1.97%, 13.99%)	1.48	0.140
	Lithotomy position	82.18%						
Moderate perineal tears (%)	Lateral position	11.34%	−6.37% (−14.97%, 2.22%)	−1.45	0.146	−6.01% (−13.99, 1.97%)	−1.48	0.140
	Lithotomy position	17.82%						
Severe perineal tears (%)	Lateral position	0%	—	—	—			
	Lithotomy position	0%						
Episiotomy (%)	Lateral position	5.15%	−4.09% (−12.33%, 4.14%)	−0.98	0.329	−5.14% (−12.37%, 2.09%)	−1.39	0.164
	Lithotomy position	13.20%						
2‐h postpartum bleeding (ml)	Lateral position	300.00 ± 100.37	80.74 (43.04, 118.42)	4.20	< 0.001***	73.97 (33.14, 114.80)	3.55	< 0.001***
	Lithotomy position	218.37 ± 122.83						
2‐h postpartum bleeding > 500 mL (%)	Lateral position	3.26%	4.45% (−2.18%, 11.08%)	1.31	0.189	−0.32% (−4.98%, 4.35%)	−0.13	0.894
	Lithotomy position	1.32%						
Spontaneous vaginal birth (%)	Lateral position	100%	—	—	—	—	—	—
	Lithotomy position	99%						
Duration of second stage of labour (min)	Lateral position	23.85 ± 20.92	0.920 (−4.04, 5.88)	0.36	0.716	0.859 (−5.09, 6.81)	0.28	0.777
	Lithotomy position	26.29 ± 24.28						
Prolonged second stage of labour (%)	Lateral position	3.09%	−0.66% (−3.83%, 2.52%)	−0.41	0.685	−0.23% (−4.99%, 4.54%)	−0.09	0.925
	Lithotomy position	1.99%						
Neonatal outcomes								
Apgar score < 7 (1 min)	Lateral position	0%	—		—			
	Lithotomy position	0.33%						
Apgar score < 7 (5 min)	Lateral position	0%	—		—			
	Lithotomy position	0%						

*** *p* < 0.001.

** *p* < 0.01.

* *p* < 0.05.

### 3.4 ATE and ATT of Lateral Positions on Secondary Outcomes

Lateral positions were associated with a significantly greater amount of blood loss at 2 h postpartum among both primiparous women (ATE: 73.09, 95% CI: 44.84101.34; *p* < 0.001) and parous women (ATE: 80.74, 95% CI: 43.04118.42; *p* < 0.001). However, lateral positions did not significantly affect the proportion of women with blood loss exceeding 500 mL at 2 h postpartum, regardless of parity (*p* > 0.05, respectively).

In this study, more women who adopted lateral positions had spontaneous births, regardless of parity. However, because very few parous women experienced assisted birth, statistical analyses of the mode of birth could not be performed.

Lateral positions were not associated with prolonged duration of the second stage of labour among the overall population (*p* > 0.05, respectively). However, among primiparous women who used lateral positions, the second stage of labour was significantly longer by 10 min (ATT: 10.39, 95% CI: 2.66, 18.12; *p* = 0.008).

No cases of neonatal Apgar scores below seven at 1 or 5 min after birth were reported in the matched groups; therefore, no relevant statistical analysis could be performed. Detailed maternal and neonatal outcomes are shown in Tables 4 and 5.

## 4 Discussion

This study analysed the effects of lateral positions and lithotomy positions during the second stage of labour on maternal and neonatal outcomes between matched groups using PSM analysis. The current study found that lateral positions could have protective impacts on the perineum among primiparous women, demonstrated by a significantly reduced risk of episiotomy use (ATE: 21.49%, *p* = 0.003), an increased likelihood of mild perineal tears (ATE: 13.59%, *p* = 0.085) and a reduced probability of moderate perineal tears (ATE: −13.59%, *p* = 0.085). However, the use of lateral positions throughout the second stage of labour did not promote spontaneous vaginal birth among primiparous women, as the ATE of lateral position was insignificant (ATE: −1.68%, *p* = 0.563). Lateral positions may prolong the duration of the second stage of labour of primiparous women who actually adopted lateral positions (ATT: 10.39, *p* = 0.008).

Additionally, it should be noted that both primiparous and parous women in lateral positions may experience a greater amount of blood loss at 2 h postpartum than those in lithotomy positions (*p* < 0.05), despite the fact that these two groups did not differ statistically in the proportion of women losing more than 500 mL of blood (*p* > 0.05). This study could not examine the impacts of lateral positions on the neonatal Apgar scores at 1 and 5 min postpartum, given the rare incidences of Apgar scores less than seven. A larger sample size or pooled data from similar studies may help clarify whether lateral positions could improve neonatal outcomes.

Perineal trauma is a common maternal morbidity during childbirth. Given that Asian ethnicity is a risk factor for perineal trauma (Quist‐Nelson et al. 2017), Chinese women are at high risk. Reducing perineal trauma and protecting the perineum during childbirth are important for Chinese women. In this study, the use of lateral positions throughout the second stage of labour was associated with a significant 21% reduction in episiotomy use among primiparous women. Episiotomy is generally used to facilitate birth and reduce severe perineal trauma, but it also causes the same extent of injury to the perineum as a second‐degree perineal tear. In addition, a non‐significant 13.59% increase in mild perineal tears, as well as a non‐significant 13.59% reduction in moderate perineal tears, was also found among primiparous women. The protective effects were even greater among primiparous women who actually adopted lateral positions in this study, as demonstrated by a significantly reduced risk of moderate perineal tears (ATT: −14.51%, *p* = 0.022). Moderate or severe perineal trauma tends to have more profound adverse impacts on women than mild perineal trauma, including perineal pain, anal incontinence, dyspareunia, greater risk of haemorrhage and negative emotions (Cattani et al. 2022; Ende et al. 2021). Taken together, findings from this study suggest lateral positions might prevent perineal tears, especially among primiparous women.

The results of this study are consistent with several other studies. A Belgian retrospective study found that fewer episiotomies were performed among women adopting lateral positions (6.7%) during childbirth compared to those in lithotomy positions (38.1%) (*p* < 0.001) (Meyvis et al. 2012). Additionally, lateral positions were associated with a 47% increased likelihood of having an intact perineum after adjusting for confounders (OR: 0.53; 95% CI: 0.36, 0.78). However, this study did not specify the timing of adopting lateral positions during childbirth. A quasi‐randomized controlled study involving 712 primiparous Chinese women reported significantly reduced rates of second‐degree perineal tears (13.8% vs. 20.0%, *p* < 0.01), episiotomy (4.1% vs. 34.0%, *p* < 0.00) and increased rates of intact perineum (28.7% vs. 14.9%, *p* < 0.01) and first‐degree perineal tears (52.5% vs. 29.4%) among women who adopted lateral positions throughout the second stage of labour compared to those who gave birth in lithotomy positions (Wei et al. 2017). It should be noted that this study reported only similar maternal age, gestational age and foetal weight between the two groups. Other possible confounders were not reported or compared. Due to the rare incidence of third‐ and fourth‐degree perineal tears, statistical analyses could not be performed in this study. A previous Swedish cohort study reported that lateral positions implied a slightly reduced risk of third‐ and fourth‐degree perineal tears in primiparous women, but the comparator was a sitting position (Elvander et al. 2015).

Several reasons could explain the overall better perineal outcomes of women giving birth in lateral positions. In this position, the perineum could stretch more gradually, and the pressure of the foetal head on the perineum is reduced (Zang, Lu, Zhang, et al. 2020). Lateral positions could also help avoid rapid expulsion of the foetal head, thereby reducing possible perineal trauma. Midwives could more easily apply hands‐on manoeuvres to protect the perineum when women adopt lateral positions.

In the current study, lateral positions did not increase the risk of blood loss greater than 500 mL at 2 h postpartum, as no significant statistical difference was detected between women who adopted lateral and lithotomy positions during childbirth (*p* > 0.05). A novel finding of this study is that women in lateral positions during the second stage of labour had a greater amount of blood loss at 2 h postpartum than those in lithotomy positions. De Jonge et al. (2007) suggested that uterine atony and pressure on the arterial and venous sides could be the physical explanations for the increased blood loss in upright positions compared to supine positions. However, the physical mechanisms underlying the increased blood loss in lateral positions compared to lithotomy positions are not clear.

Previous studies reported no significant difference in blood loss at 2 h postpartum between those adopting lateral and lithotomy positions during childbirth (Guo et al. 2015; He and Liu 2017). The discrepancy in results may be attributed to differences in participant inclusion criteria and timing of adopting lateral positions between the previous studies and the current study. Overall, based on the findings from this study, midwives should be cautious about the potential for increased postpartum blood loss when supporting women giving birth in lateral positions. Further research is needed to determine whether such an increase in blood loss at 2 h postpartum has clinical significance.

Evidence on the impacts of lateral positions on reducing assisted vaginal births is limited. In this study, fewer primiparous women in lateral positions had assisted vaginal births compared with those in lithotomy positions, suggesting lateral positions may have the potential to promote spontaneous vaginal births. One frequently discussed advantage of lateral positions is their potential to enhance the rotation of an occiput posterior foetus (Bueno‐Lopez et al. 2018). Occiput posterior positions are associated with an increased risk of Caesarean birth and assisted vaginal births (Barth 2015). Therefore, lateral positions might increase the possibility of spontaneous vaginal births by facilitating the rotation of occiput posterior positions. However, the effect of lateral positions on the rotation of an occiput posterior position is not yet certain, as existing evidence shows inconsistent results (Bueno‐Lopez et al. 2018; Desbriere et al. 2013; Le Ray et al. 2016). Furthermore, the incidence of occiput posterior position in this study was low. The effects of lateral positions on promoting spontaneous vaginal births still warrant further studies with a larger sample size.

The ATE for the duration of the second stage of labour and rates of the prolonged second stage of labour did not differ significantly between women adopting lateral positions and those adopting lithotomy positions, indicating that lateral positions may not prolong labour across the entire study population. However, among primiparous women who actually adopted lateral positions, these positions could have prolonged their duration of the second stage of labour by 10 min (ATT: 10.39, *p* = 0.008). The findings from the current study also indicated that lateral positions may not be advisable if labour is progressing slowly. Other studies also reported non‐significant differences in the duration of the second stage of labour between women in lateral and lithotomy positions (Guo et al. 2015; Wei et al. 2017). However, the effects of lateral positions on the duration of the second stage of labour are somewhat controversial. A randomized controlled trial involving 173 primiparous women found that lateral positions during childbirth could shorten the duration of the second stage of labour by 8 min compared to lithotomy positions (*t* = 2.422, *p* = 0.018) (He and Liu 2017). It is commonly assumed that lateral positions allow for better pelvic outlet expansion, which could facilitate labour progress by allowing the foetal head to descend more easily. More robust biomechanical studies are needed to confirm whether lateral positions actually change pelvic diameters.

Additionally, lateral positions should be applied cautiously in low‐resourced settings with insufficient staffing, given the potential for slow labour progress. Prolonged labour not only leads to reduced maternal satisfaction and negative birth experiences (Gaudernack et al. 2020; Kempe and Vikström‐Bolin 2020) but may also increase the workload for the obstetric staff.

Due to the rare incidence of an Apgar score of less than seven in this study, relevant statistical analyses could not be performed. In recent years, improvements in the quality of care have greatly enhanced neonatal outcomes, which may explain the low incidence of Apgar scores of less than seven in this study. It is hypothesized that lateral positions could help avoid uterine compression of the aorta and/or the inferior vena cava, thereby ensuring adequate oxygen supply to the foetus (Gupta et al. 2017). Conversely, intra‐abdominal vessels might be compressed if women assume lithotomy positions during childbirth (Huang et al. 2019). However, the beneficial effects of lateral position on neonatal outcomes remained uncertain. A randomized controlled study involving both primiparous and parous women (Guo et al. 2015) and a quasi‐experimental study recruiting primiparous women (Wei et al. 2017) both reported non‐significant differences in the rates of neonatal asphyxia between women in lateral positions and those in lithotomy positions. Future studies with a larger sample size are needed to explore the effects of lateral positions on neonatal outcomes.

Overall, this study provides further insights into the use of lateral positions for both primiparous and parous women in clinical practice. Midwives are encouraged to support women in adopting lateral positions when there are signs of perineal trauma, as these positions may offer protective benefits for the perineum. However, midwives should closely monitor blood loss and labour progression when women are in lateral positions during the second stage of labour. Although this study sheds light on the effects of lateral positions on maternal and neonatal outcomes, further qualitative or mixed‐method research exploring women's subjective experiences of adopting lateral positions during childbirth would add valuable perspectives.

### 4.1 Strengths and Limitations

This multicentre observational study adopted a rigorous study design and outcome measurements. The use of PSM as a robust methodological approach effectively controlled for multiple confounders that have not been considered in previous studies. Additionally, the standardized midwifery practices among participating hospitals also helped minimize relevant bias. We analysed the effects of lateral position by parity, which facilitates a better understanding of lateral positions across different populations.

However, this study also has several limitations. Firstly, the sample was determined based on rates of moderate perineal tears, which means it may not be sufficiently powered to detect significant differences in rarer outcomes, such as severe perineal tears and neonatal Apgar scores less than seven. Thus, the non‐significant findings should be interpreted with caution. Secondly, due to the observational nature of the study design, a causal relationship between lateral positions and maternal and neonatal outcomes cannot be definitively established. Thirdly, an inherent limitation of PSM is that only known covariates are included in estimating the propensity scores. There might be some unknown covariates, including individual midwife preferences, institutional protocols and socio‐cultural factors influencing maternal position choices, that were not identified or included in this study. Lastly, despite efforts to improve data quality through midwife training, some data were missing regarding both the characteristics of women and the maternal and neonatal outcomes, which might influence the results. However, the proportion of missing data is very small overall.

## 5 Conclusion

This study provides further guidance on the use of lateral positions in both primiparous and parous women in clinical practice. Based on PSM analyses, this observational study found that lateral positions may reduce episiotomy use among primiparous women. Additionally, lateral positions had greater protective impacts on the perineum for women who adopted them in this study. Overall, lateral positions appeared to be relatively safe for use in both primiparous and parous women, as they were not associated with prolonged labour and blood loss greater than 500 mL at 2 h postpartum. However, this study did identify a greater amount of blood loss in lateral positions compared to those in lithotomy positions. Thus, midwives should be mindful of blood loss when assisting women giving birth in lateral positions. They should also exercise caution when supporting women in lateral positions if labour progresses slowly, especially among primiparous women.

Given the rare cases of an Apgar score less than seven, this study could not assess the effects of lateral positions on neonatal outcomes. Nevertheless, it suggests implications that future studies could include a larger sample size to better evaluate the effects of maternal positions on neonatal Apgar scores.

These results also suggest that midwifery education should place greater emphasis on clinical judgement in supporting lateral positions and on careful monitoring of labour progress and postpartum blood loss. Maternity units may consider updating maternal position guidelines to reflect these safety considerations while promoting informed choice.

## Author Contributions

**Jing Huang:** conceptualization, methodology, software, formal analysis, writing – original draft. **Hong Lu:** conceptualization, methodology, supervision, writing – review and editing. **Jianying Wang:** resources, supervision. **Minghui Yang:** resources, investigation. **Yinchu Hu:** writing – review and editing. **Xue Feng:** resources, investigation. **Lihua Ren:** investigation. **Yu Zang:** investigation. **Rui Hou:** writing – review and editing, supervision.

## Funding

The authors have nothing to report.

## Ethics Statement

This study obtained ethical approval from the Institutional Review Broad of Peking University (IRB approval number: IRB00001052‐2004).

## Consent

Written consent was obtained from each participant before data collection.

## Conflicts of Interest

The authors declare no conflicts of interest.

## Data Availability

The data of this study can be made available by the corresponding author upon reasonable request.
